# Effects of RTV coating on the electrical performance of polymer insulator under lightning impulse voltage condition

**DOI:** 10.1371/journal.pone.0187892

**Published:** 2017-11-14

**Authors:** Farah Adilah Jamaludin, Mohd Zainal Abidin Ab-Kadir, Mahdi Izadi, Norhafiz Azis, Jasronita Jasni, Muhammad Syahmi Abd-Rahman

**Affiliations:** Centre for Electromagnetic and Lightning Protection Research (CELP), Faculty of Engineering, Universiti Putra Malaysia, UPM, Serdang, Selangor, Malaysia; Beijing University of Posts and Telecommunications, CHINA

## Abstract

Located near the equator, Malaysia is a country with one of the highest lightning densities in the world. Lightning contributes to 70% of the power outages in Malaysia and affects power equipment, automated network systems, causes data losses and monetary losses in the nation. Therefore, consideration of insulator evaluation under lightning impulses can be crucial to evaluate and attempt to overcome this issue. This paper presents a new approach to increase the electrical performance of polymer insulators using a Room Temperature Vulcanisation (RTV) coating. The evaluation involves three different settings of polymer insulator, namely uncoated, RTV type 1, and RTV type 2 upper surface coatings. All the insulators were tested under three different conditions as dry, clean wet and salty under different impulse polarities using the even-rising test method. The voltage breakdown for each test was recorded. From the experiment, it was found that the effectiveness of the RTV coating application became apparent when tested under salty or polluted conditions. It increased the voltage withstand capabilities of the polymer insulator up to 50% from the basic uncoated insulator. Under dry and clean conditions, the RTV coating provided just a slight increase of the breakdown voltage. The increase in voltage breakdown capability decreased the probability of surface discharge and dry band arcing that could cause degradation of the polymeric material housing. The RTV type 1 coating was found to be more effective when performing under a lightning impulse. The findings might help the utility companies improve the performance of their insulators in order to increase power system reliability.

## Introduction

Polymer insulators have been widely in used in the energy distribution industry and electrical utilities for more than four decades now. It has gained the attention of researchers and utilities due to its advantages such being lightweight, has a low installation cost, ease of handling, vandalism resistance and most importantly its high performance under pollution conditions due to its hydrophobic characteristics [1–2]. Nevertheless, throughout the years of service, utilities and researchers have found some drawbacks of using polymer insulators such as aging and degradation. From previous studies, most of the researchers found that electrical and environmental stresses were the main factors that contributed to the aging of polymer insulators. Electrical stress such as leakage current causes the formation of dry band arcing and a lightning impulse could cause flashover. On the other hand, environmental stresses such as UV radiation, heat, humidity and pollution were found to be the contributing factors to polymer material degradation and aging. The presence of accumulated pollution on an insulator surface can became conductive when wetted and thus allow the flow of a leakage current. The hydrophobic characteristic of a polymer insulator helps to bead water on the surface. However, Joule heating from a leakage current will cause certain areas to become dry and this can cause dry band arcing. If the arcing or discharge is strong enough, it can cause a flashover across the insulator [3]. In addition, since the structure of a polymer insulator consists of different materials such as polymeric housing, FRP rod and metal end fittings, interference between these materials makes polymer insulators prone to electrical deterioration. Based on previous studies [4–7], the degradation of polymeric insulators causes a loss of its hydrophobic characteristic, surface flaking, cracks, erosion, punctures on the shed or housing and worst of all it allows moisture to penetrate and affect the insulator core.

The RTV coating application method has been widely used for porcelain or glass insulators in reducing the probability of flashover compared with other methods due to its good dielectric properties, flexibility over a wide range of temperatures, adhesion characteristics, improved immunity to de-polymerisation, faster application and most importantly the application can be done under energised conditions [8–10]. One of the major advantages of the RTV silicone coating is its ability in retaining water repellence under outdoor weathering and high voltage conditions. With a clean insulator surface, RTV with a low surface energy property does not allow wetting on the insulator surface. On the other hand, when the insulator surface is contaminated, the RTV low molecule weight silicone fluid that diffuses from the bulk of the coating creates a monolayer of fluids (prevents the contaminant from dissolving in water) and imparts a non-wetting property/hydrophobicity to the contaminant layer. This results in the formation of a weak and non-conductive electrolyte layer, which is not conducive to the development of a leakage current or flashover [11–12].

From previous research, studies of RTV coatings have only reviewed glass or porcelain types of insulator. Reference [11][13] mentioned that RTV coating applications on ceramic insulators can last for up to 15 years. Furthermore, RTV coatings can be applied direct to an energised insulator with less maintenance needed which makes it the best alternative coating method compared to a grease coating [14]. However, as far as is known, no past measurements have been made for an RTV coated polymer insulator especially under a lightning impulse condition. Evaluation of insulator performance under a lightning impulse is crucial due to high-density of lightning occurrences in Malaysia. Therefore, the aim of this paper is to investigate the effects of an RTV coating on a polymer insulator in order to improve insulator performance under lightning impulse conditions and standard wave shapes.

## Methodology

Experimental work was used to investigate the breakdown voltage under a lightning impulse test. In Malaysia, 70% of power outages are attributed to lightning [15] which causes surge overvoltage on the insulator and could damage the insulator itself and trigger failure of the overall power system [16]. Failure of a power line system could cost downtime or power losses and monetary losses for the power utilities. Therefore investigation of insulator withstand capabilities under a lightning impulse is crucial to ensure reliability of the power line system in Malaysia.

Lightning is a sudden electrostatic discharge that normally occurs during rainstorms. Rainstorms produce three types of lightning, namely, intra-cloud, cloud to cloud and cloud to ground lightning. The most frequent type of lightning is cloud to cloud. However, cloud to ground lightning can be hazardous for the power line systems. Cloud to ground lightning can be either positive or negative lightning. Negative lightning occurs when the negative charges in the cloud are moving towards the ground and this is the most common form of cloud to ground lightning. On the other hand, positive lightning takes place when a positively charged cloud creates a positive streamer that discharges into the negatively charged ground. Despite the low probability of occurrence, positive lightning is more hazardous than negative lightning as it carries ten times the charge and voltage. Therefore, consideration of both negative and positive lightning is crucial to evaluate the behaviour of an insulator under different impulse polarities in order to fully evaluate insulator performance and hence improve power system reliability.

**Fig 1:**
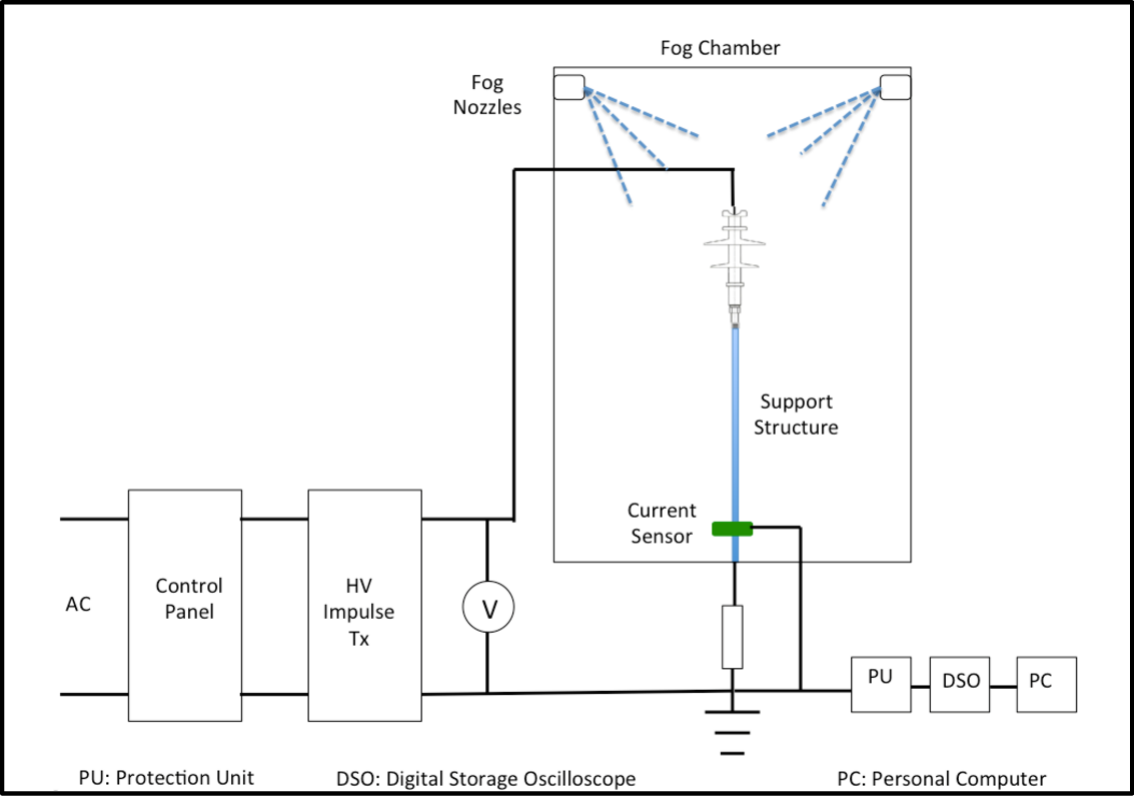
Experimental setup.

**Fig 2:**
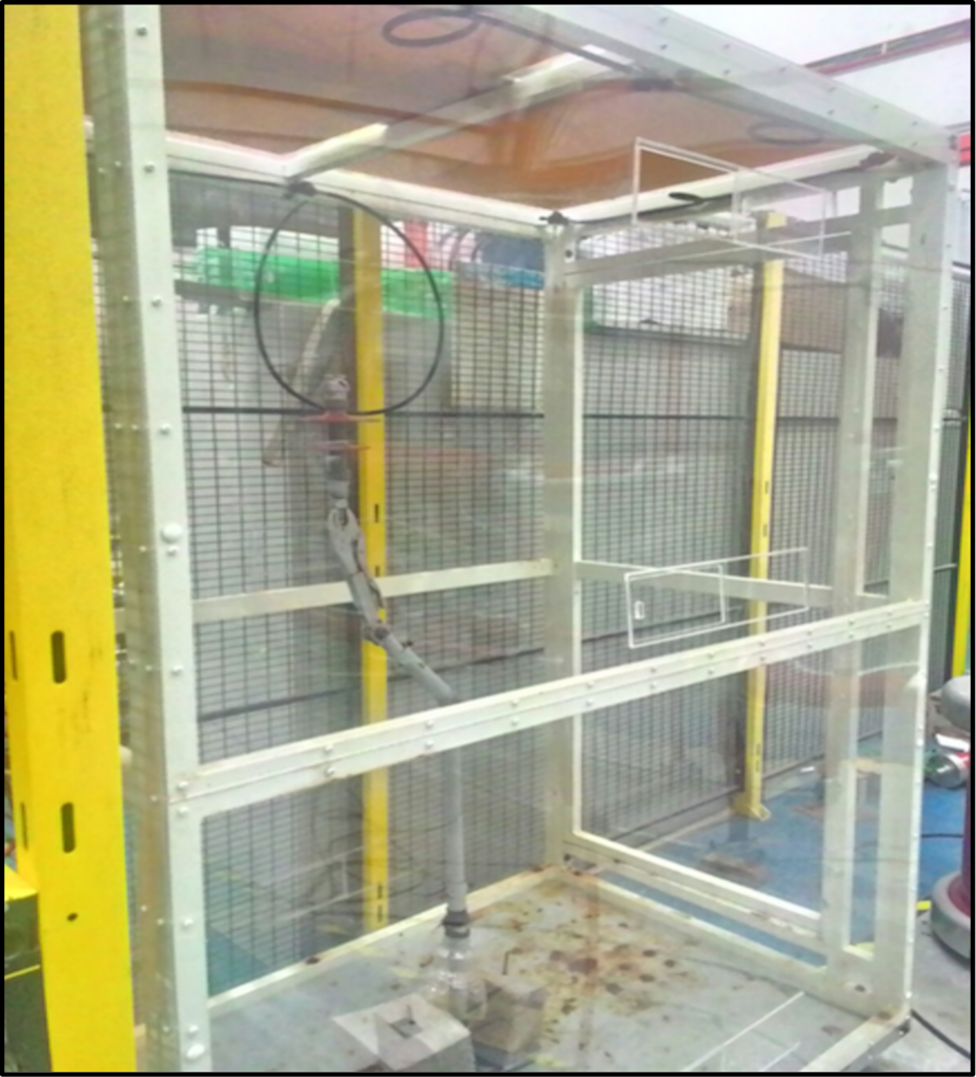
Insulator tested inside fog chamber.

Lightning impulse tests were conducted under three different conditions, dry, clean-wet and with pollution. For the purpose of this test, a 10 kV polymer type insulator was used as a specimen. Description of polymer insulator used is tabulated in Table 1. The specimens were divided into three configurations, namely, basic uncoated, RTV Type 1 and RTV Type 2 upper surfaces coated. The experimental setup is shown in Fig 1. Each specimen was placed inside a 1.5x1x2m fog chamber as shown in Fig 2. The fog chamber was equipped with six fog nozzles that produced a maximum mist rate of 48 L/h and were fabricated according to IEC507 standards [17]. For the lightning impulse test, the insulator was energised with a standard lightning impulse voltage of 1.2/50 μs, as per IEC 60060–1 standard [18]. The impulse voltage was generated using an Impulse Generator with front time of 1.2 μs ±30% and tail time of 50 μs ±20%, as defined by the standard. A high sampling rate data acquisition system (DAS) with a sampling rate of 1 GSa/s was used to capture the voltage and current and a digital storage oscilloscope (DOS) was used to visualise the voltage and current. A still digital single-lens reflex (DSLR) camera was placed in mutually perpendicular directions to capture the arc paths during the flashover events.

**Table 1:** Polymer insulator geometric details (manufacturer’s details).

Rated Voltage (kV)	10
Rated Mechanical Load (kN)	4
Min Arcing Distance (mm)	165
Min Nominal Creepage Distance (mm)	420

**Fig 3:**
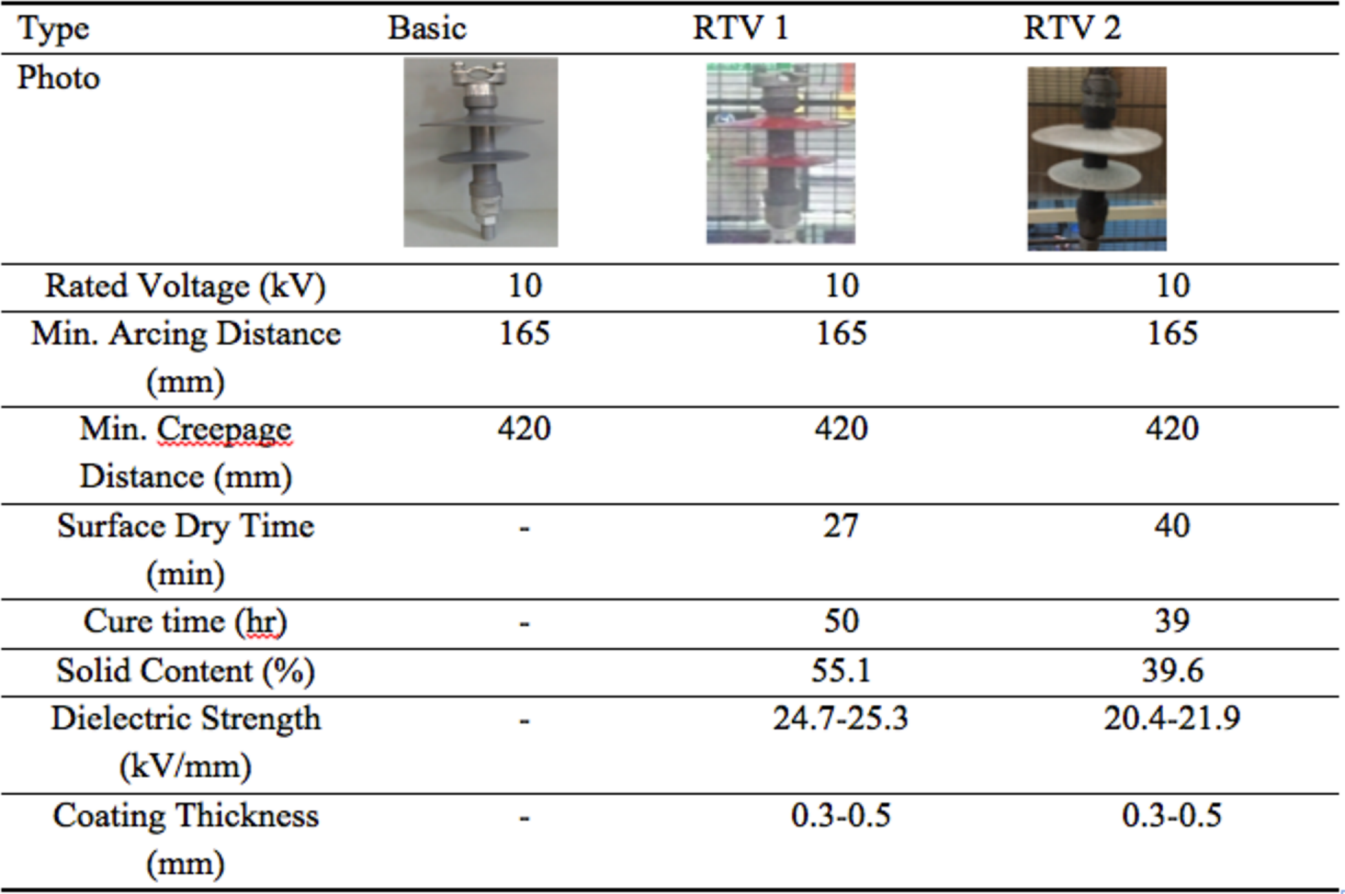
Parameters of test specimen.

Two types of RTV coating were applied to the insulators, namely, RTV 1 and RTV 2 as shown in Fig 3. The differences of these two types of coating materials were depending on their formulations that determine their physical and electrical properties. These properties were depending on type of polymer, fillers and the amount of free fluid in the coating during its manufacturing process. The selection of these RTV coating were based on its electrical parameters and material properties. RTV type 1 was using high-grade nano-material while RTV type 2 was using normal material. Details properties of RTV coatings used are tabulated in Table 2.

**Table 2:** Technical specifications of RTV coating materials (manufacturers’ details).

Properties	RTV 1	RTV 2
Surface Dry Time (minutes)	27	40
Cure Time (hours—at room temperature)	50	39
Solid Content (%)	55.1	39.6
Dielectric Strength (kV/mm)	24.7–25.3	20.4–21.9
Tensile Strength (mpa)	3.951	1.9
Shear Strength (mpa)	3.574	1.991
Tear Strength (kN/m)	15.2	9.4
Durable years (outdoors)	15	5–8

RTV coating was applied on to the upper surface of the insulator with the aid of fine paint brush. The insulators were clean and allowed to dry before coating. The thickness of the coating was between 0.3 to 0.5 mm [19]. After coating, the insulators were leave to cure to between 39 to 50 hours according to manufacturer’s details as stated in Fig 3. The coating configuration was chosen based on cases where pollution accumulated on the upper surface of the insulator which caused an increase in the surface resistance and increase the surface temperature due to the leakage current [9]. Estimated cost to apply RTV coating material were tabulated as in Table 3. The estimated costs were based on 1 span double circuit distribution tower or pole. From the estimated cost, additional RTV coating on polymer insulator will increase the cost of insulator from 10.9% to 16.5%.

**Table 3:** Estimated cost of RTV coating material on polymer insulator.

	RTV 1 (USD)	RTV 2 (USD)
Cost of RTV per insulator (max 20mil.)	3	2
Cost of each polymer insulator	18.2	18.2
No of insulators used for double circuit	6	6
Cost per 1 span	127.2	121.2

In Malaysia, installations of insulators are located at various locations such as in coastal areas, high land, and low land. The pollution at each location can be different depending on the type of development surrounding the installation areas. The types of pollution can be from a variety of sources such as sea salt, carbon, sand, acids and many more. However, the most common pollution is produced from sea salt and carbon. Therefore, for this study, sea salt was chosen to be the source of pollution. For testing under pollution conditions, salt fog was applied to the insulators. The pollution was replicated by mixing distilled water and 40 g of sodium chloride (NaCl) to produce a 4% Equivalent Salt Deposit Density (ESDD) as in the IEC 60507 standards. In order to observe the performance of the insulator with or without an RTV coating, the withstand test according to the IEC 60060–1 standard was followed. All the test specimens were tested under positive and negative impulses. During impulse testing, the even-rising test method was adopted to obtain the breakdown voltage. The purpose of this method was to determine 50% of the voltage breakdown probability (U50). In this method, the r.m.s voltage was set at the minimum voltage available and increased at a rate of 5 kV/min until a breakdown occurred. A new test was repeated five minutes after each breakdown for 20 times on the same insulator.

## Results and discussion

There are several factors that determine the occurrence of flashover when the insulator or power line has been struck by lightning. These factors include the wave shape and polarity of the lightning surge, the withstand characteristics of the insulator and the power frequency component of the voltage across the insulator [16]. Therefore, investigating the insulator breakdown strength under a lightning impulse is important to increase knowledge of insulator performance under lightning stress and to promote the reliability and stability of the power lines.

An Impulse Lightning test was conducted under different conditions in order to determine the behaviour of the breakdown voltage of the insulator. The purpose of conducting the test under dry conditions was to set a base or reference value. The clean-wet test was conducted under clean-fog conditions in order to ensure that the specimen was totally covered by wetness and under high humidity conditions. Under polluted conditions, the salt-fog test method was chosen to test the specimen, as it was relatively easy to prepare and was a suitable method to ensure uniformity of the pollution. Application of an RTV coating on the upper surface of the insulator was chosen because of its material properties and it was easy to apply on the insulator. The RTV coating was applied to the insulator in order to protect the polymer shed from deterioration and to increase its withstand capabilities in order to improve insulator performance under lightning stress.

### Voltage at breakdown

The normal procedure for determining the lightning withstand voltage is based on the even-rising method whereby the determination of 50% probability of the flashover, U_50_ required at least 20 tests or shots, as per IEC 60060–1 standard. During the test, voltage is increased every 5kV steps until breakdown occurred where the next test will be repeated every 5 minutes. Therefore, the number of measurements in a test series should be at least 20 times to ensure accurate results obtained. To observe the effects of the RTV coating on the surface of the polymer insulator, a comparison of data was made between two types of RTV surface coated insulators with a third basic uncoated insulator. Table 4 shows the result of the U50 for the basic uncoated insulator tested under dry, clean-wet and polluted conditions. From the table, the U50 value was highest under the dry condition regardless the polarity of the impulse, while under the clean-wet condition the U50 of the insulator decreased up to 56.5% with a negative impulse and slightly decreased by about 2.69% with a positive impulse. This was due to the moisture on the insulator surface when wetted which increases the surface conductivity of the insulator at the pre-breakdown stage. Therefore the U50 under the clean-wet condition was slightly lower when compared to the insulator tested under the dry condition. On the other hand, under the polluted condition the U50 for the basic uncoated insulator was tremendously decreased by about 45.1% with a positive impulse and 60.7% with a negative impulse. The massive reduction was due to the formation of a conductive layer on surface of the insulator that allowed the flow of a leakage current and discharges on the surface.

**Table 4:** Basic uncoated insulator impulse test.

*Polarity*	*Condition*	*U50 (kV)*	*Std.dev (kV)*
Positive	Dry	200.8	5.03
	Clean Wet	195.4	3.58
	Salt	110.3	11.82
Negative	Dry	256.9	6.77
	Clean Wet	111.6	5.50
	Salt	100.9	7.88

Table 5 shows the results for the RTV type 1 surface coated insulator tested under different conditions. From the experiment, it shows that the U50 for the RTV type 1 surface coated was highest under the dry condition regardless of impulse polarities. Under the clean wet condition, the percentage difference of the U50 when compared to test conducted under the dry condition was 2.27% with a positive impulse and 35.5% with a negative impulse. This was for the same reason as explained in Table 4 above. The test conducted under the polluted condition showed a reduction of the U50 up to 24.3% with a positive impulse and 36.9% with a negative impulse. The pollution layer on the insulator surface, when introduced with water formed a conductive layer on the surface of the insulator. This allows a flow of leakage current on the surface and when the amplitude was high, strong discharges could occur which could lead to flashover.

**Table 5:** RTV type 1 surface coated insulator impulse test.

*Polarity*	*Condition*	*U50 (kV)*	*Std.dev (kV)*
Positive	Dry	229.2	4.01
	Clean Wet	224.0	4.67
	Salt	173.6	15.21
Negative	Dry	248.7	8.26
	Clean Wet	160.5	11.46
	Salt	157.0	7.48

Table 6 shows the tabulated results for the RTV type 2 surface coating when tested with a lightning impulse. The U50 for the RTV type 2 coated insulator was highest under the dry condition regardless the impulse polarity. The percentage difference from the U50 tested under the clean wet condition was 2.81% with a positive impulse and 15.9% with a negative impulse. The differences were due to the increase of surface conductivity under the wet condition. For the polluted condition, the U50 decreased by about 36.6% with a positive impulse and 54.0% with a negative impulse when compared with the U50 under the dry condition. The justification of the reduction has been explained for Tables 4 and 5 above.

**Table 6:** RTV type 2 surface coated insulator impulse test.

*Polarity*	*Condition*	*U50 (kV)*	*Std.dev (kV)*
Positive	Dry	234.6	3.77
	Clean Wet	228.0	7.86
	Salt	148.8	6.69
Negative	Dry	265.3	7.27
	Clean Wet	223.0	10.07
	Salt	122.0	6.57

From the tabulated results in Tables 4 to 6, under the dry condition the application of the RTV type 1 or type 2 on the polymer insulator surface did not show much difference compared to the basic uncoated insulator. The percentage of difference for RTV type 1 was 14.1% and RTV type 2 was 16.8%, which was higher compared to the uncoated insulator with a positive impulse. With a negative impulse the percentage difference for both RTV type 1 and 2 coating were 3.19% and 3.26% respectively compared to the uncoated insulator. Under the clean wet condition when tested with a positive impulse, the U50 for the RTV coated insulators showed percentage difference of 14.6% and 16.7% respectively from the value of basic uncoated insulator. However, with a negative impulse the percentage difference from the basic uncoated insulator slightly increased for both types of RTV coating with a percentage of 43.8% for the RTV type 1 coating and 49.9% for the RTV type 2 coating. On the other hand, under the polluted condition, the RTV type 1 coating showed a 57.39% increase in the U50 compared to the uncoated insulator and a 14.29% higher U50 compared to the RTV type 2 coating with a positive impulse polarity. However the performance with a negative impulse indicated that the U50 of the RTV type 1 coating showed 55.6% higher compared with the uncoated insulator and 28.69% higher than the RTV type 2 coating. Tables 4 to 6 summarises all the results obtained for the U50 after 20 tests. In terms of standard deviation, the highest value was 15.21 kV and the lowest value was 3.58 kV. Even after many tests were conducted, it seemed that the test results still indicated some dispersion, which may due to different frequencies of breakdown occurrence.

### Positive and negative lightning impulses

Due to fatalities and impacts of lightning impulses, testing under both polarities is necessary in order to determine the insulator withstand capabilities.

**Fig 4:**
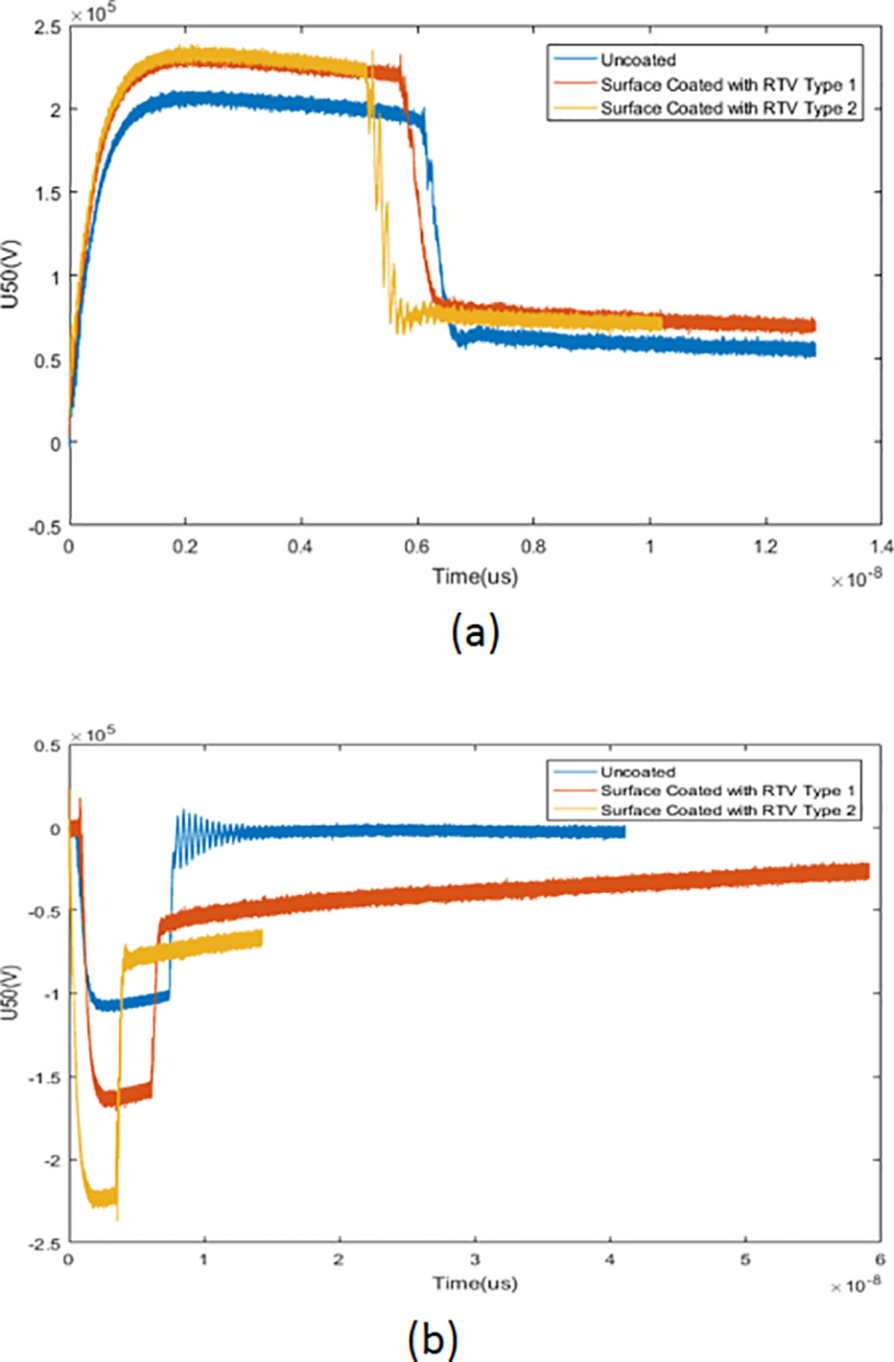
Breakdown value for clean insulator under different impulse polarities. (a) Breakdown value for clean insulator under positive impulse (b) Breakdown value for clean insulator with a negative impulse.

Fig 4 below shows the breakdown behaviour under clean wet condition tested with different impulse polarities. Fig 4A shows the breakdown behaviour with a positive impulse. From the figure, the U50 for the basic uncoated insulator was the lowest with a value of 195.4 kV compared with RTV 1 and RTV 2 surface-coated insulators with values of 224 kV and 228 kV respectively. On the other hand, Fig 4B shows the breakdown behaviour with a negative impulse. From the figure, the U50 for the basic uncoated insulator shows a value of 111.6 kV, while the RTV 1 and RTV 2 surface-coated insulators show a breakdown value of 160.5 kV and 223 kV respectively. The U50 for the RTV type 1 surface-coated insulator shows no significant difference with different impulse polarities, while for the others the breakdown value U50 was higher with a positive impulse. The breakdown voltage under the clean condition is slightly higher when tested with a positive impulse due to the migration of conductive ions from the electrode onto the insulator surface that caused high electrical conductivity and a high leakage current [20].

**Fig 5:**
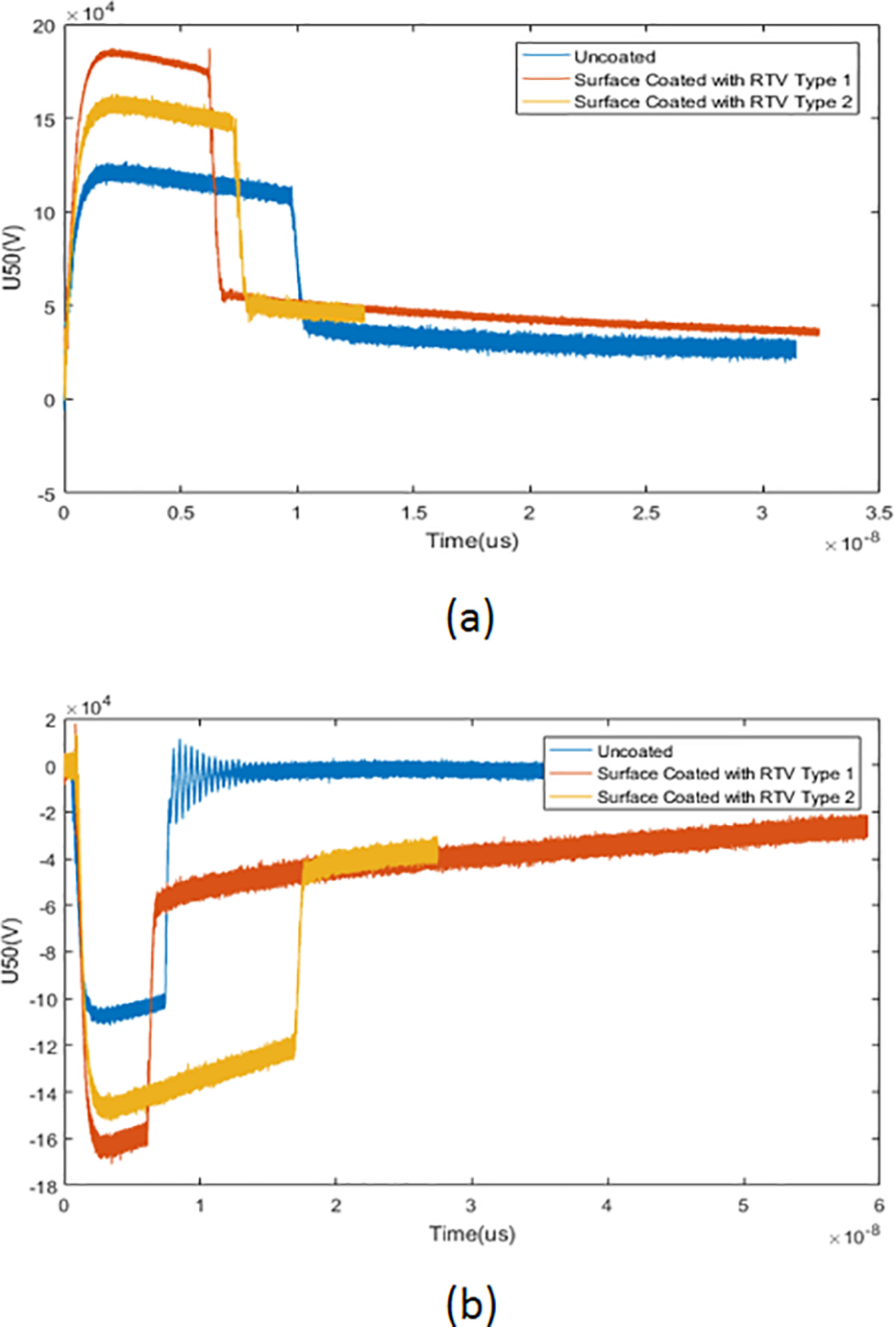
Breakdown value for polluted insulator under different impulse polarities. (a) Breakdown value for polluted insulator under positive impulse (b) Breakdown value for polluted insulator with a negative impulse.

Fig 5 on the other hand shows the breakdown behaviour under salt or polluted conditions. Fig 5A shows the breakdown behaviour with a positive impulse. From the figure it can be seen that the U50 for the basic uncoated insulator was the lowest among all the results with a value of 110.3 kV, while RTV type 1 and RTV 2 surface-coated insulators showed slightly higher breakdown values of 173.6 kV and 148.8 kV respectively. 5b shows the breakdown behaviour when tested with a negative impulse. The U50 for the basic-uncoated insulator shows a value of 100.9 kV, while RTV type 1 and RTV 2 surface-coated insulators show slightly higher breakdown values of 157 kV and 122 kV respectively. From the experiment, the breakdown value tested with a positive impulse shows slightly higher when compared to the negative impulse with a percentage difference from 4% to 16%. The dry bands depending on the electrical strength of the insulators with impulse voltages may have influenced these results [21]. Theoretically, with a negative impulse, positive charges build up in the vicinity of a high voltage negative electrode as the electrons in the gap spread towards the positive electrode. These phenomena will result in a reduction of the electric field over the major part of the gap, which in turn increases the flashover voltage. However for this study, the breakdown voltage was mostly higher with a positive impulse. Atmospheric factor and ambient temperature were the main factors affecting the experimental results. This may also due to space charge formation around the high voltage electrode, caused by an electron attachment process. The water-bridged sheds may also influence the reverse polarity effects and finally the same space-charge effects may contribute to an increase of flashover voltage under a positive impulse [22]. These phenomena also explain in reference [23] that relate the reduction of breakdown voltage under negative impulse might be due to loss of hydrophobicity on insulator surfaces and also due to flashover conduction through continuous water path rather than through air.

From both Figs 4 and 5 a difference of time duration during breakdown was indicated. From both figures, regardless of the test conditions, the breakdown duration for basic uncoated insulator was slightly longer than breakdown duration for the RTV coated insulators. Based on the assumptions, the roughness of the surface coatings may be one of the contributing factors to the difference in time duration during breakdown. The surface roughness lead to accumulation of contamination on the surface and contributed to time reduction during the breakdown event [9]. From the experiment, it can be concluded that the RTV coating was more effective under the salt or pollution conditions. It showed a significant percentage difference of up to 50% compared to the basic uncoated insulator. Therefore, further investigation and concern was focused on the pollution condition. Table 7 below shows the leakage current value under the salt condition. The values of the leakage current of the RTV coated insulators were much lower than the value for the basic uncoated insulators with a percentage difference of up to 90%. The higher amplitude of leakage current may damage the polymeric material of the insulator because it carries a high temperature. This may cause material degradation and premature aging of the polymer insulator.

**Table 7:** Leakage current value under salt condition.

	Salt Condition		
	Uncoated (A)	RTV 1 (A)	RTV 2 (A)
Positive Impulse	48.5	3.46	3.06
Negative Impulse	40.5	2.39	2.12

### Surface discharge characteristics

**Fig 6:**
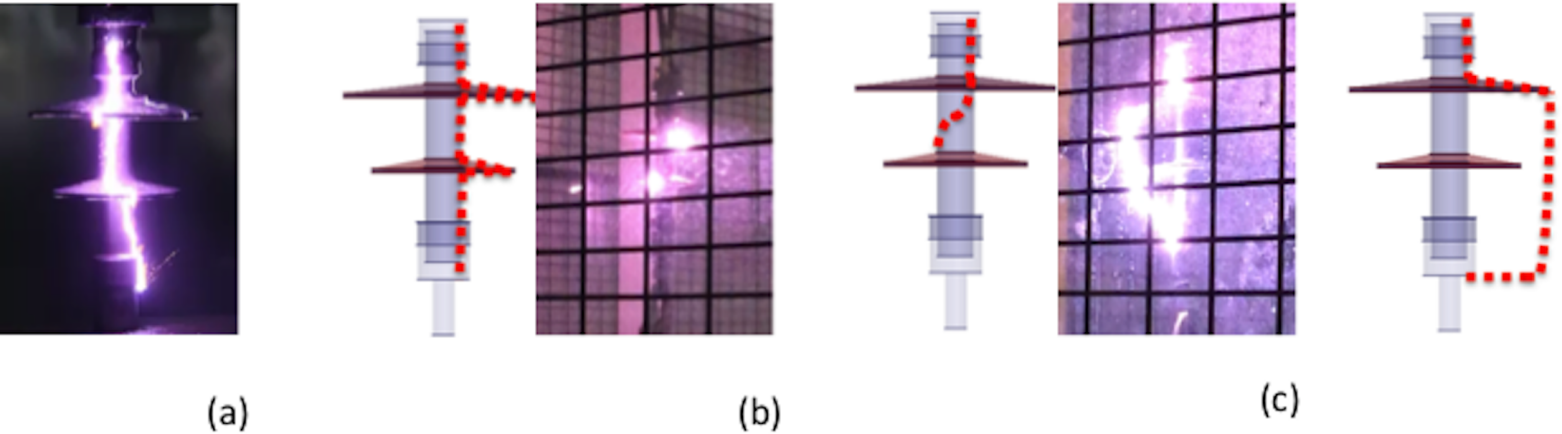
Examples of arcing path under polluted conditions. (a) Along the insulator surface (b) Spiral and halfway along insulator (c) In the air.

The arcing paths of the discharge channel were observed by using a high-speed camera as shown in Fig 6. From the experimental work, it was observed that there were four different types of channels that take place during the discharge. These were along the insulator surface, spirally along the insulator surface, halfway along the insulator and lastly, in the air. From the figure it can be seen that the non-linearity of the arcing path depended on the value of the conductivity of the insulator surface. Under the polluted condition, the surface conductivity was high due to the conductivity of the pollution layer.

The observation of arcing paths can be summarised as given in Table 8. From the table, it can be concluded that:

1. Under the dry condition, the discharge path was normally in the air because of the absence of surface conductivity on the insulator.
2. Under the clean wet condition, some discharge paths were in the air and some on the insulator surface. This was due to wetting which caused the insulator surface to become conductive and initiate surface tracking and arcing. The hydrophobicity of the RTV material helped to bead water on the insulator surface, and when heated it created a dry band and therefore determined the arcing path that normally leapt out at the HV triple junction and discharged at the nearest path whether through the air or along the insulator surface.
3. Under the polluted condition, the deposited salt on the insulator when wetted created a conductive layer on the insulator surface, thus allowing a flow of leakage current. The leakage current caused a surface discharge on insulator and affected the arcing path.
4. As explained in (ii) above, the hydrophobicity of the material contributed in determining the arcing path.
5. In addition, the arcing path may be affected by the static charge that contributed to the localisation of the electric field, especially near to HV electrodes and edges. These arcing paths will help researchers to identify critical points where the electric field was localised. Ageing, degradation and damage of the polymer material strongly depend on the arcing path and therefore utility companies should identify if the material used for the insulators is spark-phobic or otherwise [24].

**Table 8:** Arcing paths of insulators under different conditions.

	Basic uncoated	RTV Type 1	RTV Type 2
Dry Condition	Majority of the discharge paths were in the air		
Wet Condition	Some of the discharge paths were in the air and some along the insulator surface		
Salt Condition	Majority of the discharges took place along the insulator surface	Discharges could take place along the insulator surface, spirally or halfway along the insulator.	Discharges could take place in the air, along the insulator surface, spirally or halfway along the insulator.

From the studies conducted, surface wetting and pollution affected the U50 of the insulator. With the RTV coating application on the insulator upper surfaces, it can help to increase surface resistivity and reduce surface temperature, hence increasing its voltage breakdown strength when exposed to a lightning impulse. The RTV coating type 1 was found to more effective in increasing the U50 of the polymer insulator regardless the conditions of the test and impulse polarities. It can be concluded that properties of the RTV material coating such as the hydrophobic characteristic plays an important role in optimising the performance of the insulator under wet and pollution conditions due to the ability to resist the formation of a conductive layer and therefore resist the flow of leakage current on the insulator surface. It aids the insulator to perform better under electrical stress such as lightning impulse voltages.

## Conclusion

Based on the experimental work carried out on three different configurations of the polymer insulators, namely, basic uncoated, RTV type 1 surface coated and RTV type 2 surface coated, the following conclusions can be drawn:

1. Under the dry condition, the percentage difference of the U50 was not significant between uncoated and RTV coated as the percentage difference was less than 5%.
2. Under the clean wet condition, the U50 of RTV coated insulator was slightly higher compared to the basic uncoated insulator with a percentage difference of up to 20%.
3. The RTV coatings were more effective under the polluted condition. It increased the U50 value of the insulator by up to 50% regardless of the impulse polarity.
4. The RTV type 1 coating showed better performance compared with the RTV type 2 coating under the polluted condition. Material composition and high dielectric strength value might be the factor RTV type 1 shows better performance under polluted condition. From the study, the application of the RTV coating was found to be effective in terms of strengthening the voltage withstand capabilities under a lightning impulse. The RTV coating can be used in order to improve and protect the surface condition of a polymer insulator. This may help to improve the performance of the polymer insulator and increase its lifespan and power system reliability.
